# Huge liver tumor in young man

**DOI:** 10.1002/jgh3.12234

**Published:** 2019-08-02

**Authors:** Osamu Shibata, Kenya Kamimura, Rie Azumi, Kei Shibuya, Shintaro Shiba, Tatsuya Ohno, Shuji Terai

**Affiliations:** ^1^ Division of Gastroenterology and Hepatology, Graduate School of Medical and Dental Sciences Niigata University Niigata Japan; ^2^ Department of Gastroenterology and Hepatology Kameda Daiichi Hospital Niigata Japan; ^3^ Gunma University Heavy Ion Medical Center Maebashi Japan

**Keywords:** carbon‐ion radiotherapy, cholangiocarcinoma, imaging, positron emission tomography, radiation therapy

## Abstract

Intrahepatic cholangiocarcinoma is the second leading primary hepatic tumors, accounting for 5% of all hepatic tumors. The curability depends on the operability; however, the difficulty of early diagnosis and late clinical presentation account for the poor prognosis. Therefore, development of a novel therapeutic option and a method to determine the viability of the primary tumor, which hinder the assessment of the impact of other therapies, including chemotherapy and radiotherapy are needed. Although FDG–PET has been used to detect distant metastases of ICC, which are present in 20% of patients at the initial diagnosis, little is known about the efficacy of FDG–PET of the primary lesion of ICC. Here, we present the case of a 31‐year‐old male diagnosed with unresectable ICC and successfully treated with carbon‐ion radiation, and present the usefulness of fluorodeoxyglucose–positron emission tomography in the determination of the viability of the tumor.

## Graphical abstract

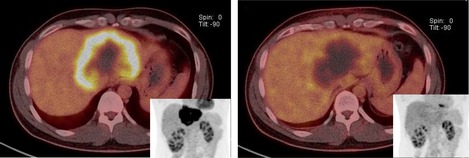

Effect of carbon‐ion radiotherapy on unresectable intrahepatic cholangiocarcinoma

## Introduction

Intrahepatic cholangiocarcinoma is the second leading primary hepatic tumors, accounting for 5% of all hepatic tumors. Currently, development of a novel therapeutic option and a method to determine the viability of the tumor, which hinder the assessment of the impact of other therapies, including chemotherapy and radiotherapy are unmet needs. Here, we present the case of a 31‐year‐old male diagnosed with unresectable intrahepatic cholangiocarcinoma and successfully treated with carbon‐ion radiation, and present the usefulness of fluorodeoxyglucose–positron emission tomography in the determination of the viability of the tumor.

## Case report

**Figure 1:**
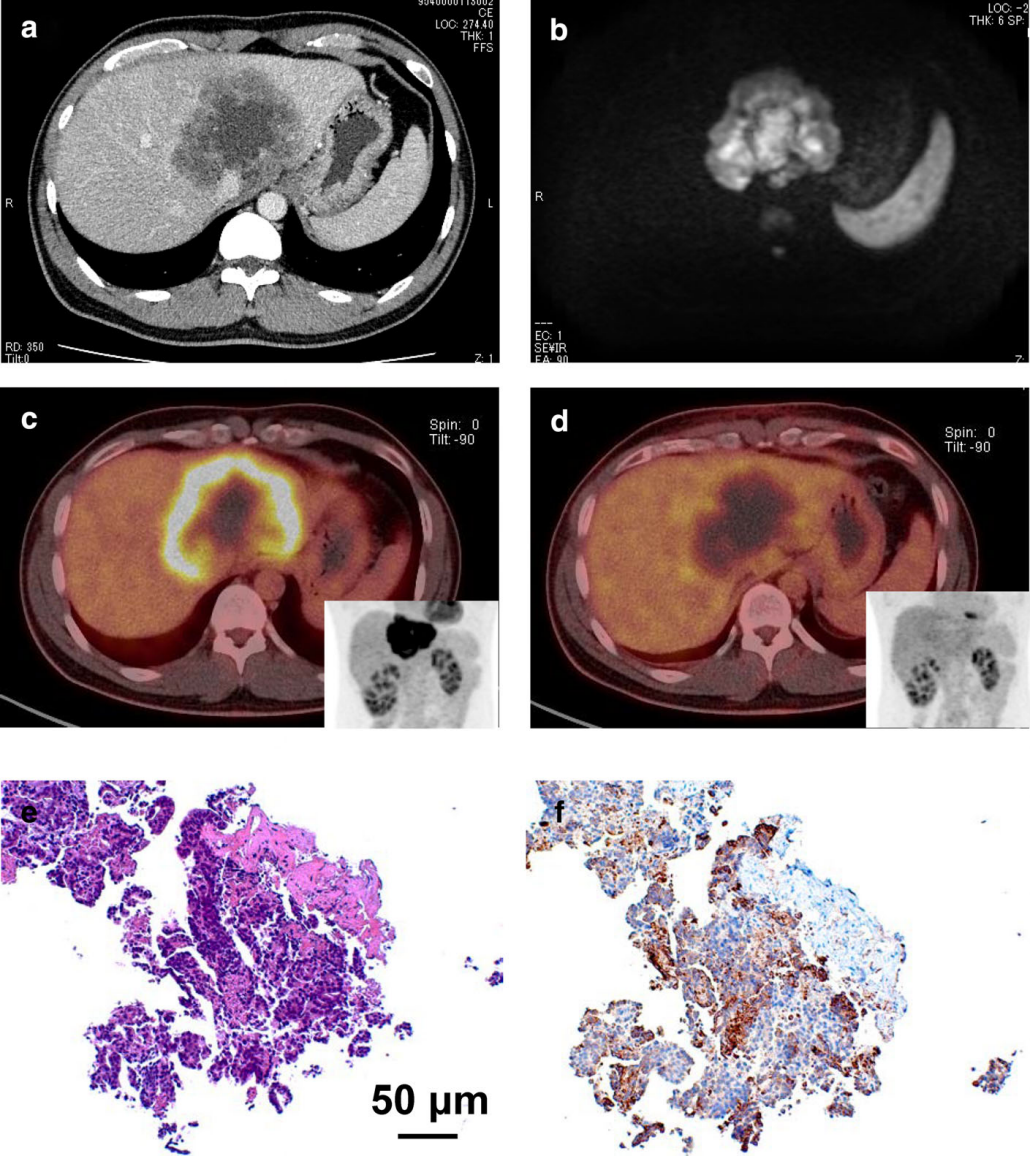
(a) Computed tomography of the tumor. Computed tomography demonstrated an 80 mm‐thick lobulated tumor in the liver adjacent to the inferior vena cava and left and right hepatic veins with a low‐density lesion in its interior and mild enhancement of the tumor rim. (b) Diffusion‐weighted images of magnetic resonance imaging. The tumor showed a high‐intensity signal pattern with diffusion‐weighted images. (c) Fluorodeoxyglucose‐positron emission tomography image before the carbon ion radiotherapy. (d) FDG‐PET image, 4 months after the carbon ion radiotherapy. (e) HE staining of the tumor tissue. (f) Cytokeratin 7 staining of the tumor tissue.

A 31‐year‐old male was referred to our hospital for further assessment of a huge liver tumor. He had no medical or family history, and laboratory examinations demonstrated no abnormal findings. Computed tomography showed an 80 mm‐thick lobulated tumor in the liver adjacent to the inferior vena cava and left and right hepatic veins, with a central low‐density lesion and mild enhancement of the tumor rim (Fig. 1a). The tumor showed a high‐intensity signal pattern with T2‐ and diffusion‐weighted images (Fig. 1b) using magnetic resonance imaging. The central part of the tumor showed higher signals, which we suspected to be the mucus component. Fluorodeoxyglucose–positron emission tomography (FDG–PET) demonstrated a ring‐like, high and intense FDG uptake with a high‐level standardized uptake value (SUV) of 9.15 ± 1.09 (mean ± SD) at the rim of the tumor and no uptake at the center of the tumor (Fig. 1c). No other uptake was seen. Histological analysis was performed, and because of its inoperability, we performed carbon ion radiotherapy of 64.8 Gy, which significantly decreased the cell viability, represented by an SUV of 1.25 ± 0.19 (Fig. 1d), 4 months after the completion of the therapy.

Histological analysis performed on a specimen obtained from the tumor rim exhibited adenocarcinoma cells, partly forming malignant glands with fibrous tissue (HE staining, Fig. 1e). The tumor cells were positively stained with cytokeratin 7 (Fig. 1f) and hepatocyte nuclear factor‐4α and were negative for cytokeratin 20. These findings indicated the tumor to be an intrahepatic cholangiocarcinoma (ICC), which is the second leading primary liver cancer.^1^

## Discussion

The curability of ICC depends on the operability; however, early diagnosis is difficult and contributes to the poor prognosis. An additional consideration is the effect of therapies, such as chemotherapy and radiotherapy, on the viability of the primary tumor. Although FDG–PET has been used to detect distant metastases of ICC, which are present in 20% of patients at the initial diagnosis, little is known about its efficacy in determining the viability of the primary lesion.^2^ Our patient exhibited a high level of SUV before therapy, indicating a poor prognosis,^2^ which markedly decreased after carbon ion radiotherapy. Reportedly, carbon ion radiotherapy is safe and effective in various malignancies, including hepatocellular carcinoma.^3^ A prospective study comparing conventional therapy is currently ongoing. As carbon ion radiotherapy enables focused delivery of the maximum energy to the tumor, it is considered to be ideal for both target conformity and sparing of the healthy liver compared with photon‐based stereotactic body radiotherapy.^3^ Careful follow‐up has shown that the viability of the tumor was successfully controlled for a year.
